# A comparison of the optical method with the mechanical method in routine coagulation tests

**DOI:** 10.5937/jomb0-56100

**Published:** 2025-06-13

**Authors:** Gözde Ülfer

**Affiliations:** 1 Istanbul Medipol University, Faculty of Medicine, Department of Biochemistry, Istanbul, Türkiye

**Keywords:** coagulation tests, optic method, mechanic method, Bland-Altman plots, Passing-Bablok regression, testovi koagulacije, optička metoda, mehanička metoda, Bland-Altman grafici, Passing-Bablok regresija

## Abstract

**Background:**

This study aimed to compare the prothrombin time (PT), international normalised ratio (INR) and activated partial thromboplastin time (aPTT) values obtained using the photo-optical method and to assess these values according to the reference method, which was the mechanical method.

**Methods:**

Plasma samples from 340 patients, submitted to our hospital's biochemistry laboratory for PT, INR, and aPTT analyses, were assayed using the mechanical coagulometric measurement method in a Stago Compact Max3 automated coagulation analyser, which served as the reference device. The same samples were also analysed using the Sunbio UP5500 automated analyser with a simultaneous optical method. There were 30 turbid samples analysed in both devices without exclusion from the study. Correlation coefficient analysis was carried out using SPSS to assess intervariable correlations. Passing-Bablok regression analysis was performed in R software version 3.6.0 to compare PT, INR, and aPTT values between the two devices. Bland-Altman plots were used to analyse the agreement.

**Results:**

A good level of statistically significant agreement was found between the PT and INR values measured by the Stago Compact Max3 and Sunbio UP 5500 devices (Interclass Coefficient Correlation (ICC): 0.627, p=0.001; p<0.01 and ICC: 0.653, p=0.001; p<0.01, respectively). Additionally, there was an excellent level of statistically significant agreement for the aPTT values (ICC: 0.902, p=0.001, p<0.01). The Bland-Altman analysis revealed the mean 95% limits of agreement values as 2.46 (lower limit: -2.44, upper limit: 7.37) for PT, 0.07 (lower limit: -0.32, upper limit: 0.46) for INR, and 2.45 (lower limit: -1.67, upper limit: 6.58) for aPTT. The Passing-Bablok regression results indicated a systematic difference for PT measurement but no proportional difference. No systematic or proportional differences were found for the measured INR and aPTT values between the Stago Compact Max3 and Sunbio UP 5500 devices. The intra-assay and interassay coefficient of variation (CV) values from level 1 and 2 controls of the optical method were below 5%.

**Conclusions:**

The results from the optical method were consistent and reliable compared to the mechanical method. PT and INR results showed statistically good agreement, while aPTT results demonstrated excellent agreement. Larger multicenter studies are needed to evaluate turbid samples.

## Abstract (sr)

**Uvod:**

Ova studija je imala za cilj da uporedi vrednosti protrombinskog vremena (PT), me|unarodnog normalizovanog odnosa (INR) i aktiviranog parcijalnog tromboplastinskog vremena (aPTT) dobijene foto-optičkom metodom i da proceni ove vrednosti u odnosu na referentnu, mehaničku metodu.

**Metode:**

Plazma uzorci 340 pacijenata su poslati u biohemijsku laboratoriju naše bolnice na analizu PT, INR i aPTT, i ispitani su mehaničkom koagulometrijskom metodom merenja na automatskom koagulacionom analizatoru Stago Compact Max3, koji je poslužio kao referentni uređaj. Isti uzorci su takođe analizirani pomoću automatskog analizatora Sunbio UP5500 sa istovremenom optičkom metodom. Trideset zamućenih uzoraka je analizirano na oba uređaja bez isključivanja iz studije. Analiza koeficijenta korelacije izvršena je korišćenjem SPSS-a za procenu korelacija izme|u promenljivih, a Passing-Bablok regresiona analiza je izvedena u softveru R, verzija 3.6.0, za poređenje PT, INR i aPTT vrednosti između dva uređaja. Za analizu saglasnosti su korišćeni Bland-Altman grafici.

**Rezultati:**

Utvrđen je dobar nivo statistički značajne saglasnosti između PT i INR vrednosti izmerenih na uređajima Stago Compact Max3 i Sunbio UP5500 (koeficijent intraklasne korelacije (ICC): 0,627, p=0,001; p<0,01 i ICC: 0,653, p=0,001; p<0,01, respektivno). Pored toga, za aPTT vrednosti postignut je izuzetan nivo statistički značajne saglasnosti (ICC: 0,902, p=0,001, p<0,01). Bland-Altman analiza je pokazala prosečne 95% granice saglasnosti sa vrednostima od 2,46 (donja granica: -2,44, gornja granica: 7,37) za PT, 0,07 (donja granica: -0,32, gornja granica: 0,46) za INR i 2,45 (donja granica: -1,67, gornja granica: 6,58) za aPTT. Rezultati Passing-Bablok regresione analize ukazali su na sistematsku razliku za merenje PT, ali bez proporcionalne razlike. Nisu pronađene sistematske ili proporcionalne razlike za merenje INR i aPTT vrednosti između uređaja Stago Compact Max3 i Sunbio UP5500. Koeficijenti varijacije (CV) unutar serije i između serija za nivoe 1 i 2 kontrole optičke metode bili su ispod 5%.

**Zaključak:**

Rezultati optičke metode su se pokazali doslednim i pouzdanim u poređenju sa mehaničkom metodom. PT i INR rezultati pokazali su statistički dobru saglasnost, dok su aPTT rezultati pokazali izuzetnu saglasnost. Potrebne su veće multicentrične studije za evaluaciju zamućenih uzoraka.

## Introduction

Prothrombin time (PT), activated partial thromboplastin time (aPTT), and international normalised ratio (INR) are among the coagulation tests frequently conducted in routine laboratories [1] [2]. PT evaluates extrinsic and common pathway functions and is influenced by the levels of factors V, VII, and X, as well as prothrombin and fibrinogen levels. This is particularly important for monitoring oral anticoagulants [3]. In contrast, aPTT assesses intrinsic and common pathway functions and is affected by levels of high-molecular-weight kininogen, prekallikrein, and factors VIII, IX, XI, and XII [4] [5]. Routine coagulation tests employ two different technological systems based on mechanical and optical clot detection methodologies. However, the question of which method is superior remains debated, highlighting the need for further studies.

It is widely accepted that turbid samples do not influence mechanical clot detection, thus considered superior to photo-optical methods, which may be affected by turbidity [6] [7] [8] [9]. This perception supports the belief that mechanical clot detection provides a more accurate determination of clotting time (CT) for coagulation testing. However, several studies suggest that optical and mechanical clot detection methods yield equivalent results in correlation, accuracy, and precision and that both methods are unaffected by sample turbidity [10] [11] [12].

This study aimed to compare the PT, INR, and aPTT values obtained using the mechanical method with those obtained through the photo-optical method. It is the first study to compare the new optical system, Sunbio UP5500, with the reference device, Stago Compact Max3. Thus, the results will contribute to the literature regarding comparing these two methods.

## Materials and methods

Approval for this study was secured from the Ethics Committee of Istanbul Medipol University (approval number: E-10840098-202.3.02-6034, date: October 4, 2024). We included all patients who were healthy and were using anticoagulant therapy in the study. Plasma samples from a total of 340 patients, submitted to our hospital’s biochemistry laboratory for PT, INR, and aPTT analyses, were assayed using the mechanical coagulometric measurement method in the Stago Compact Max3automated coagulation analyser, which served as the reference device. The same samples were then analysed using the Sunbio UP5500 automated analyser with the simultaneous optical method. Turbid samples were analysed in both devices without exclusion from the study. HIL (hemolysis, icteric, lipemia) indices were measured on Roche Cobas c 503, with Sample Index Gen. 2 kit. There were 30 turbid samples. The comparison was conducted over a six-day period.

Plasma was prepared by centrifugating blood samples at 3,000 rpm for 10 minutes in citrated tubes, and the mechanical coagulometric measurement method was employed in the Stago Compact Max3 automated coagulation analyser to measure PT, INR, and aPTT. PT was measured using an STA NeoPTimal kit (Lot number: 271081), while aPTT was measured using an STA C.K PREST kit (Lot number: 271038). The PT test relies on calcium thromboplastin to measure the clotting time from the patient’s plasma and compare it to the normal standard. In contrast, the aPTT test involves re-coagulating plasma with a standard amount of cephalin and kaolin. INR was calculated using the ratio of the patient’s PT to the mean of the normal reference range, elevated to the strength of the reactive international sensitivity index (ISI). The ISI for the PT reagent used was 1, and the calculation followed the formula, INR= (PTpatient/PTmean normal)^ISI^.

The same samples were also analysed using the Sunbio UP5500 automated coagulation analyser with the simultaneous optical method. The test principle, based on coagulation, chromogenic, and immunoturbidimetric methods, produced results using PT and aPTT reagents to analyse plasma coagulation, anticoagulation, fibrinolysis, and antifibrinolytic functions. The Sunbio Determination of Prothrombin Time kit (Lot number: 8001E536) was used for PT, while the Sunbio Determination Activated Partial Thrombo plastin Time kit (Lot number: 8011E2761) was used for aPTT. The ISI for the PT reagent used was 1.04, and the following formula was used for the calculation: INR=(PTpatient/PTmean normal)^ISI^. The assay precision was assessed using internal quality controls (Lot number: 182E045), with level 1 and 2 control results repeated 20 times intra-assay. Inter-assay controls for levels 1 and 2 were done 3 times a day, and the comparison continued over six days [13].

Calibration and control samples were assayed following the manufacturer’s standard methods. These tests were evaluated using normal and abnormal controls daily and participating in a monthly External Quality Control of Diagnostic Assays and Tests program for the reference device.

### Statistical analyses

The Statistical Package for the Social Sciences (SPSS) version 27 was used for statistical analyses of the study results. Quantitative variables were presented using mean, standard deviation, median, minimum, and maximum values. The Shapiro-Wilk test and box plots were employed to evaluate the conformity of the data to a normal distribution. An interclass coefficient (ICC) correlation analysis assessed intervariable correlations. A Passing-Bablok regression analysis was performed in R software version 3.6.0 to compare PT, INR, and aPTT between the two devices. Bland-Altman plots were used to analyse the agreement. Post hoc power analyses were performed for the study. The results were evaluated at a 95% confidence interval with a significance level of p<0.05.

## Results

The agreement analysis between the PT, INR, and aPTT values measured on the Stago Compact MAX3 and Sunbio UP5500 devices (n=340) is shown in Table 1.

**Table 1:** Agreement analysis between PT, INR, and aPTT values measured on Stago Compact MAX3 and Sunbio UP5500 devices (n=40).

		Stago Compact Max3	Sunbio UP5500	ICC	p	Slope 95% CI	Intercept 95% CI
PT	Median + SD	14.93 ± 3.62	12.47 ± 1.93	0.627	** 0.001 **	0.60 (0.57/0.63)	3.59 (3.12/4.05)
	Median (Q1–Q3)	14.2 (13.3–15.3)	12.1 (11.6–12.8)				
INR	Median + SD	1.11 ± 0.29	1.04 ± 0.17	0.653	** 0.001 **	0.66 (0.63/0.70)	0.31 (0.28/0.35)
	Median (Q1–Q3)	1.1 (0.98–1.14)	1 (0.97–1.07)				
aPTT	Median + SD	29.63 ± 4.69	27.18 ± 4.84	0.902	** 0.001 **	0.93 (0.88/0.98)	-0.44 (-1.87/0.98)
	Median (Q1–Q3)	29.2 (27.5–31)	26.7 (24.6–28.75)				

**Figure 1:**
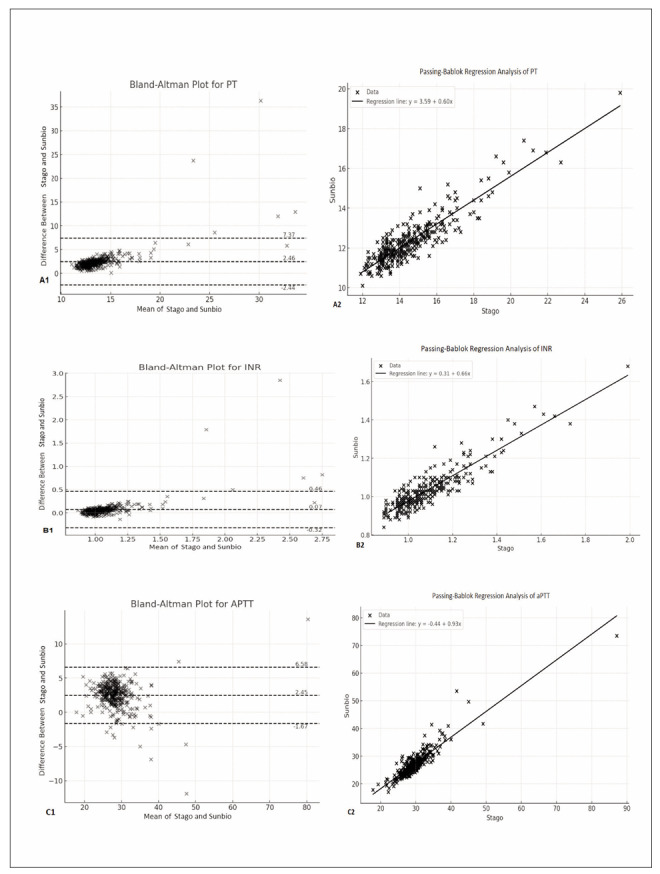
Comparison of tests by applying Bland-Altman plots and Passing-Bablok regression models. PT (A1, A2); INR (B1, B2); aPTT (C1, C2).

A good level of statistically significant agreement was established between the PT values measured by the Stago Compact Max3 and Sunbio UP5500 devices (ICC: 0.627, p=0.001; p<0.01). Post hoc power for PT was 100%. Bland Altman plots, used to assess agreement and show 95% confidence intervals for each pairwise comparison, indicated good agreement between the PT measurements from both devices. According to the Bland- Altman plot, the meanlimits of agreement (95% LoA) between the two devices for PT values were 2.46 (lower limit: - 2.44, upper limit: 7.37) (Figure 1, A1). The Passing-Bablok regression analysis indicated a systematic difference between the devices, as the PT intercept value significantly differed from 0. However, no proportional difference was observed concerning the slope value (Figure 1, A2).

For INR values, there was also a good level of statistically significant agreement between the Stago Compact Max3 and Sunbio UP 5500 devices (ICC: 0.653, p=0.001; p<0.01). Post hoc power for INR was 96.9%. Bland Altman analysis demonstrated good agreement, with a mean LoA value of 0.07 (lower limit: -0.32, upper limit 0.46) (Figure 1, B1). The Passing-Bablok regression plot indicated no systematic or proportional differences between the INR measurements of the two devices based on the intercept and slope values (Figure 1, B2).

An excellent level of statistically significant agreement was found for aPTT values between the Stago Compact Max3 and Sunbio UP 5500 devices (ICC: 0.902, p=0.001; p<0.01). Post hoc power for aPTT was 99.9%. Bland-Altman plots indicated good agreement, showing a mean 95% LoA value of 2.45 (lower limit: -1.67, upper limit: 6.58) (Figure 1, C1). The Passing-Bablok regression analysis revealed no systematic or proportional differences between the aPTT measurements of the two devices based on the intercept and slope values (Figure 1, C2).

Intra-assay precision was assessed for levels I andII across PT, INR, and aPTT measurements (Table 2). At level I, the lowest CV was observed for aPTT, followed by PT, while INR had the highest CV. Similarly, at level II, aPTT had the lowest CV, followed by PT, while INR had the highest CV. These results suggest that INR assays demonstrated the highest consistency. It was also determined that the CV values for PT, INR, and aPTT were lower at level II than level I, indicating higher reproducibility and consistency at level II, especially for PT and INR assays. As all CVs were below 5% at both levels, the test results were considered highly consistent and reliable.

**Table 2:** Intra-assay precision analyses of the measured PT, INR, and aPTT values at levels I and II (n=20).

Intra-assay precision			
		Level I	Level II
PT	* CV *	4.93%	1.25%
	* Mean ± SD *	12.11 ± 0.60	40.95 ± 0.51
INR	* CV *	4.95%	1.31%
	* Mean ± SD *	1.01 ± 0.05	3.58 ± 0.05
aPTT	* CV *	1.73%	1.06%
	* Mean ± SD *	27.14 ± 0.47	46.95 ± 0.50

Inter-assay precision was assessed for levels I and II based on PT, INR and aPTT values measured 3 times a day over six days (Table 3). At both levels, aPTT had the lowest CV, followed by INR, while PT had the highest CV. These findings indicate that INR assays maintained the highest consistency across inter-assay evaluations. The test results were deemed highly consistent and reliable, with all CVs being under 5% at both levels.

**Table 3:** Inter-assay precision analyses of the measured PT, INR, and aPTT values at levels I and II over six days (n=3).

Intra-assay precision			
		Level I	Level II
PT	* CV *	2.20	1.36
	* Mean ± SD *	12.23 ± 0.27	40.65 ± 0.55
INR	* CV *	1.94	1.32
	* Mean ± SD *	1.03 ± 0.02	3.56 ± 0.05
aPTT	* CV *	0.57	0.54
	* Mean ± SD *	26.67 ± 0.15	46.37 ± 0.25

## Discussion

This study is the first to comparetheperformance of the newly developed optical system Sunbio UP5500 system with the Stago Compact Max3, a reference device for coagulation testing. We used the Passing-Bablok regression analysis to assess the agreement between the two methods. In addition, Bland-Altman plots were used as graphical analysis to visually compare the optimal method with the reference mechanical method [14] [15]. In this study, the effect of preanalytical variables on the results was equal because the samples were assayed simultaneously. This made the analytical performance assessment more advantageous than in earlier studies. Unlike the study by Avcı et al. [16], which excluded hemolysed, lipemic, and icteric samples to prevent interference, our study included all turbid samples. It achieved consistent results despite potential sample turbidity.

Agoodlevel of statistically significant agreement was found between the PT and INR values measured by the Stago Compact Max3 and Sunbio UP5500 devices (ICC: 0.627, p=0.001; p<0.01 and ICC: 0.653; p=0.001; p<0.01, respectively). Additionally, there was an excellent level of statistically significant agreement between the aPTT measurements of the two devices (ICC: 0.902, p=0.001; p<0.01). According to the Bland-Altman plot, the mean 95% LoA value was determined to be 2.46 (lower limit: -2.44, upper limit: 7.37) for PT, 0.07 (lower limit: -0.32, upper limit: 0.46) for INR, and 2.45 (lower limit: -1.67, upper limit: 6.58) for aPTT. Passing-Bablok regression analysis demonstrated a systematic difference for the PT assay, but not proportionally, likely due to differences in the reference ranges of the kits used by each system (11–15.5 sec for Stago Compact Max3 and 10–14 sec for Sunbio UP 5500). Reference ranges become important when evaluating PT results. We think the systematic difference in PT values will not be reflected in the clinic since the follow-up is done with the INR in anticoagulant treatment with Warfarin [1]. No systematic or proportional differences were found for INR and aPTT measurements. Additionally, intra-assay and inter-assay CV values for levels 1 and level 2 in the optical method remained below 5%, indicating that the results of this method are consistent and reliable when compared to the mechanical method.

The findings obtained from the current study align with the literature. For example, Tekkesin et al. [17] reported similar performance between optical and mechanical coagulation methods (r=0.97 for PT testing and r=0.85 for PTT testing) [6]. The authors also found no significant difference when testing turbid samples. Similarly, Bai et al. [18] found high correlation coefficients for PT and aPTT (r=0.99) when comparing photo-optical and electromechanical clot detection methods. Variations in correlation coefficients may result from using different kits or device technologies. Zengi et al. [19] also reported intraassay and inter-assay CV values under 5% for the optical method and high correlation coefficients (above 0.95) for both PT and aPTT, especially for the latter (0.976). Consistent with these studies, our results demonstrated that PT and INR values showed statistically good agreement, with excellent agreement observed for aPTT measurements. Moreover, our intra-assay and inter-assay CV values below 5% further support the precision and reliability of the optical method when compared to the reference method.

Some earlier studies widely recognise that mechanical clot detection is not affected by turbid samples andis thereforeconsideredsuperior tophotooptical clot detection, which can be influenced by turbidity [6] [7] [8] [9]. The ability of new optical systems to read at different wavelengths may have impacted the results, potentially delivering equivalent performance in this study. Other studies suggest that optical and mechanical clot detection methods for coagulation testing are comparable in correlation, accuracy, and precision, and both methods are unaffected by sample turbidity [10] [11] [12]. The findings of this study support these conclusions.

### Limitations

The primary limitation of this study was the small number of turbid samples included within the overall sample pool. Larger multicenter studies are needed to evaluate turbid samples.

## Conclusion

The results of this study revealed that the optical method, when compared to the mechanical method, provided consistent and reliable results. PT and INR measurements showed statistically good agreement, while aPTT measurements demonstrated excellent agreement. In conclusion, the Sunbio UP5500 optical system’s performance was comparable to the reference mechanical system, making it a reliable and high-precision option for routine coagulation testing.

### Ethics committee approval

Approval for this study was secured from the Ethics Committee of Istanbul Medipol University (approval number: E-10840098-202.3.02-6034, date: October 4, 2024).

### Authors’ contributions

GU: Conceptualization, data curation, formal analysis, investigation, methodology, project administration, supervision, visualisation, writing – original draft, writing – review & editing.

### Financial disclosure

There is no financial disclosure in this study.

### Conflict of interest statement

All the authors declare that they have no conflict of interest in this work.
